# Sampling Design Influences the Observed Dominance of *Culex tritaeniorhynchus*: Considerations for Future Studies of Japanese Encephalitis Virus Transmission

**DOI:** 10.1371/journal.pntd.0004249

**Published:** 2016-01-04

**Authors:** Jennifer S. Lord, Hasan Mohammad Al-Amin, Sumit Chakma, Mohammad Shafiul Alam, Emily S. Gurley, Juliet R. C. Pulliam

**Affiliations:** 1 Vector Group, Liverpool School of Tropical Medicine, Liverpool, United Kingdom; 2 Department of Biology, University of Florida, Gainesville, Florida, United States of America; 3 Centre for Communicable Diseases, icddr,b, Mohakhali, Dhaka, Bangladesh; 4 Emerging Pathogens Institute, University of Florida, Gainesville, Florida, United States of America; 5 Fogarty International Center, National Institutes of Health, Bethesda, Maryland, United States of America; United States Army Medical Research Institute of Infectious Diseases, UNITED STATES

## Abstract

Mosquito sampling during Japanese encephalitis virus (JEV)-associated studies, particularly in India, has usually been conducted via aspirators or light traps to catch mosquitoes around cattle, which are dead-end hosts for JEV. High numbers of *Culex tritaeniorhynchus*, relative to other species, have often been caught during these studies. Less frequently, studies have involved sampling outdoor resting mosquitoes. We aimed to compare the relative abundance of mosquito species between these two previously used mosquito sampling methods. From September to December 2013 entomological surveys were undertaken in eight villages in a Japanese encephalitis (JE) endemic area of Bangladesh. Light traps were used to collect active mosquitoes in households, and resting boxes and a Bina Pani Das hop cage were used near oviposition sites to collect resting mosquitoes. Numbers of humans and domestic animals present in households where light traps were set were recorded. In five villages *Cx*. *tritaeniorhynchus* was more likely to be selected from light trap samples near hosts than resting collection samples near oviposition sites, according to log odds ratio tests. The opposite was true for *Cx*. *pseudovishnui* and *Armigeres subalbatus*, which can also transmit JEV. *Culex tritaeniorhynchus* constituted 59% of the mosquitoes sampled from households with cattle, 28% from households without cattle and 17% in resting collections. In contrast *Cx*. *pseudovishnui* constituted 5.4% of the sample from households with cattle, 16% from households with no cattle and 27% from resting collections, while *Ar*. *subalbatus* constituted 0.15%, 0.38%, and 8.4% of these samples respectively. These observations may be due to differences in timing of biting activity, host preference and host-seeking strategy rather than differences in population density. We suggest that future studies aiming to implicate vector species in transmission of JEV should consider focusing catches around hosts able to transmit JEV.

## Author Summary

The relative numbers of individuals of each mosquito species in an area are important to estimate when identifying species that contribute the most to vector-borne pathogen transmission. However, methods to sample mosquitoes and enumerate the number of individuals collected often vary in their catch efficacy between species. For example, species that take a bloodmeal during daylight hours are less likely to be caught using a light trap than a species that feeds predominantly at night. Similarly, sampling near a mammalian host will more likely collect mosquitoes with a preference for mammals than those with a preference for birds. In this study we compare sampling methods for assessing the relative abundance of mosquito species that may be involved in Japanese encephalitis virus (JEV) transmission. Collections near cattle- a species unable to transmit JEV- have been influential in implicating *Cx*. *tritaeniorhynchus* as the primary vector of JEV in South Asia, due to the high number of individuals of this species caught relative to other species. Indeed, this mosquito constituted the majority of the mosquitoes collected by light traps in households with cattle in this study. However, other species were more common when sampling households without cattle or resting mosquitoes near oviposition sites. We propose that methods used to sample mosquitoes in studies aiming to implicate species in JEV transmission in South Asia be reconsidered given that there are other mosquito species that are able to transmit JEV, and these species may be underrepresented when sampling using light traps near cattle.

## Introduction

Japanese encephalitis virus (JEV) is one of the most important causes of viral encephalitis in Asia with an estimated 67,900 cases annually [1]. The transmission cycle of JEV was initially studied in Japan during the 1950’s [2–7]. The most important mosquito vector—*Culex tritaeniorhynchus*—and reservoir hosts—pigs and ardeid birds—first implicated in Japan are generally thought to drive transmission across Asia [8–11].

The high numbers of *Cx*. *tritaeniorhynchus* caught during JEV-associated field studies have reinforced current theory about the primary role of this mosquito species in JEV transmission [3,12–21]. In particular, *Cx*. *tritaeniorhynchus* has often constituted the majority of the mosquitoes sampled in regions of India reporting Japanese encephalitis (JE) cases [15–17,20–36]. For this reason, in addition to observations of this species feeding predominantly on mammalian hosts, including pigs, and the number of viral isolates obtained from this species, *Cx*. *tritaeniorhynchus* is thought to be the primary vector in India [17,37]. JEV has however, been detected in 15 other mosquito species that are found in this region [37]. Although detection of virus in a mosquito species does not indicate that it is a vector, it does demonstrate that the species has fed upon a viremic host and its potential as a vector should be further considered.

Studies in India reporting high numbers of *Cx*. *tritaeniorhynchus* relative to other species have often been undertaken around cattle (dead-end hosts for JEV [38]) and, less frequently, pig sties and human dwellings [16,17,20,21,24–26,28–31,33,35]. Justification for sampling near cattle was made by reference to the ability of this method to catch large numbers of JEV vector species in China [24,39]. Outdoor resting collection methods have also been developed to collect *Cx*. *tritaeniorhynchus* in addition to other species, although these alternative methods have not been used as frequently in studies aiming to implicate vector species in JEV transmission [40]. In addition, few studies in South Asia have compared these methods with respect to the relative abundance of mosquito species caught.

Mosquito sampling methods can be biased toward certain species [41–45]. However, differences in the observed abundance of *Cx*. *tritaeniorhynchus* relative to other potential vector species between sampling methods in South Asia has been little studied. Bias in estimates of mosquito species relative abundance could have important implications for the process of identifying the host and mosquito species that drive transmission, given that the two are linked by the host-feeding preferences of the mosquito [46].

In regions of India experiencing JE outbreaks, between 85–98% of *Cx*. *tritaeniorhynchus* bloodmeals have been found to be from cattle, even when resting mosquitoes were sampled away from potential hosts [47–49]. The preference of *Cx*. *tritaeniorhynchus* for feeding on cattle has also been demonstrated under experimental conditions [50]. Thus, if other species exhibit different host preferences, using collection methods near cattle may lead to an overrepresentation of *Cx*. *tritaeniorhynchus* within mosquito samples.

We therefore aimed to compare the relative abundance of mosquitoes between two previously used mosquito sampling methods in a region of Bangladesh where JE cases have been reported [51]. More specifically, we aimed to: 1) quantify the difference in species composition of mosquitoes captured in resting collections adjacent to oviposition sites (method 1) and those captured at light traps placed in households near humans and domestic animals (method 2); and 2) quantify the association between the numbers of humans and domestic animals, including cattle, in a household and the relative abundance of common mosquito species captured at light traps.

## Materials and Methods

### Study sites

Hospital-based surveillance between 2003–2005, and 2007–2008 and related JE burden studies indicated the Rajshahi Medical College Hospital catchment area in northwest Bangladesh as having the highest rates of human JE incidence in Bangladesh [51,52]. Based on this finding, eight villages within this catchment area (Naogaon, Chapai Nawabganj and Rajshahi Districts within Rajshshi Division) were randomly selected from a census database using a random number generator (S1 Fig). These villages were surveyed during the JE transmission season [51], between September and December 2013. In each of the eight villages, village boundaries were established upon arrival by consulting with the local chairperson.

### Sampling method 1

Resting collections were made at only seven of the eight villages, due to time constraints. Collections were made for one to four days in each village, depending on the time available (S1 Table). Resting collections were conducted in shaded areas of vegetation adjacent to mosquito oviposition sites (e.g. ponds, ditches and rice fields). Ten to 20 resting boxes, constructed from 1m long metal frames covered with black refuse bags, were placed near various habitats in the chosen sites during the first day at each village and checked each morning between 8.30 and 12.00. Resting collections were also made using a Bina Pani Das (BPD) hop cage, which was constructed according to Das [40]. Using the hop cage, approximately every meter along a transect, vegetation was disturbed for 30 seconds as described by Das [40]. The number of transects and their length varied according to the shape and size of the area available for sampling, but an average of three transects of 20m in length were surveyed in each village. The action of disturbing the vegetation resulted in resting mosquitoes flying up into the cage, from which they could then be collected after each 30 second sampling period. The length of each transect and number of samples were recorded. Two surveyors checked resting boxes and used the hop cage concurrently, collecting mosquitoes by hand-held battery powered aspirators.

### Sampling method 2

From six to 12 Centers for Disease Control and Prevention miniature light traps were set per night in each village depending upon the time available (S1 Table). Light was the only attractant used with traps, which were set from dusk to dawn. Traps were set on the first night in households along the main village road so that each light trap was approximately evenly distributed from another and so that they covered the extent of the village. Traps were moved to different households upon subsequent days of trapping within a village. GPS locations in Google Earth were used to select households for the placement of the next night’s light traps to ensure traps were approximately evenly distributed.

At the time of survey, numbers of all domesticated animals and humans living in each household where light traps were set were obtained by interview with a household member. Locations where animals were kept at night varied between selected households. Locations of light traps inside household areas, including an indoor room where humans slept, animal shed, indoor areas where both humans and animals were present, and outdoor courtyards where animals were kept were therefore alternated between households to enable approximately equal sampling effort in each area. Mosquitoes were collected from light traps each morning after resting collections were made.

### Processing of mosquitoes

All mosquitoes were killed using chloroform in an open well ventilated space, and immediately separated from other insects at a local field station after every morning collection. Trained entomologists further separated female mosquitoes from males and preserved them in tubes containing silica gel and cotton wool. As there is no specific key to the mosquitoes of Bangladesh, a number of taxonomic keys [53–60] from other countries in Asia were used that collectively included the species listed by Ahmed [61]. Females, where possible, were identified to species level and numbers of each species per catch (e.g. per light trap, per group of boxes, or per BPD hop cage transect) recorded. Three of 123 light trap catches were estimated by identifying approximately one third of the catch, due to high numbers collected (>5000 female mosquitoes each).

### Data repository

All data are available in a GitHub repository along with source code for the analyses: https://github.com/PulliamLab-UFL/mosquito-Rajshahi. In addition to the eight villages where household host data were collected, light traps were also set in an additional two villages for which no household host data were collected. The data from these two villages were therefore not included in data analyses but are included in the repository.

### Data analysis

We use the term ‘relative abundance’ to describe numbers of mosquitoes caught by each sampling method in order to acknowledge that we do not know how counts per collection scales with actual population density. Common species were defined as those constituting at least 5% of the total mosquito collection by either method.

### Species composition according to sampling method

For both sampling methods, the proportion of the total catch constituting each species and approximate 95% confidence intervals (1.96*√*p*(1-*p*)/*N*, where *p* is species proportion and *N* is the total number of mosquitoes) were calculated. Estimates of species richness were compared between sampling methods, taking into consideration the number of individual female mosquitoes collected. To do this, we considered the total number of species caught as a function of the number of female mosquitoes caught by sampling method 2- the method by which the most species were obtained. This relationship was assessed via linear regression, with log (x + 1) number of female mosquitoes as the explanatory variable and species richness as the response. Regression coefficients were then used to compare species numbers across sampling methods based on the aggregated data for each method by calculating (a) the species richness expected from method 2 in a sample of 575 individuals (equivalent to the number of females obtained by method 1), and (b) the number of mosquitoes that would have been required using sampling method 2 to catch 24 species of mosquito (equivalent to the total number of species collected via method 1). Hill’s diversity numbers [62,63] were calculated for method 1 (separately for the BPD hop cage and resting boxes) and method 2 to compare sampled species diversity between the two methods.

### Comparison of common species relative abundance between sampling methods at the village-level

Potential differences in species composition between samples from each method were assessed. The probability of selecting each common species from a collection using method 2 compared to a collection using method 1 were calculated for each village where these species were collected by both methods, using log odds ratios, standard error (SE) and 95% confidence intervals for the log odds. A confidence interval that did not include zero indicated that a species was significantly more likely to be selected from a collection using method 2 than a collection using method 1 (if greater than zero) or the opposite (if less than zero).

### Effect of household host community upon the relative abundance of common species in light traps

The log (x+1) transformed count data for each common species in light traps was analyzed using multiple linear regression, as a function of the number of cattle, goats, birds and humans in households where light traps were set. All explanatory variables, including interaction terms, were initially included in the model, and then a step-wise elimination of non-significant terms according to the F-test was undertaken to achieve the minimal adequate model [64]. Using the minimal adequate model, the predict function in the R stats package [65] was used to plot the predicted relationship between individual significant explanatory variables and the number of female mosquitoes. Given the substantial effect of cattle relative to other hosts upon common species in our analysis, arithmetic means and standard error of the means (s.e.m.) of numbers of each mosquito species per light trap sample were compared for households with cattle and with no cattle.

## Results

In the eight villages where household host data were available, a total of 74,780 female mosquitoes were collected from 123 light trap nights and 32 hours of resting collections. The total sample included 36 species, belonging to the genera *Aedeomyia* Theobald, *Aedes* Meigen, *Anopheles* Meigen, *Armigeres* Theobald, *Coquillettidia* Dyar, *Culex* Linnaeus, *Mansonia* Blanchard, *Mimomyia* Theobald and *Uranotaenia* Lynch Arribalzaga (Table 1 and S2 Table). A total of 0.3% (212 of 74,205) of light trap samples had specimens that were unidentifiable due to damage.

**Table 1:** Mosquito species constituting at least 5% of resting collections near oviposition sites (method 1) or light traps near hosts (method 2).

Species	Total number caught using method 1	% of sample from method 1 (95% confidence interval)	Total number caught using method 2	% of sample from method 2 (95% confidence interval)
*Culex tritaeniorhynchus*	100	17 (14.3–20.5)	42829	58 (57.4–58.1)
*Anopheles peditaeniatus*	11	2 (0.8–3)	10281	14 (13.6–14.1)
*Culex gelidus*	58	10 (7.6–12.6)	4490	6 (5.9–6.2)
*Culex pseudovishnui*	157	27 (23.7–30.9)	4203	6 (5.5–5.8)
*Culex vishnui*	34	6 (4–7.8)	1118	1 (1.1–1.2)
*Armigeres subalbatus*	48	8 (6.1–10.6)	122	< 1 (0.1–0.2)
*Armigeres kesseli*	29	5 (3.3–6.8)	58	< 1 (0.06–0.1)

### Species composition according to sampling method

**Fig 1:**
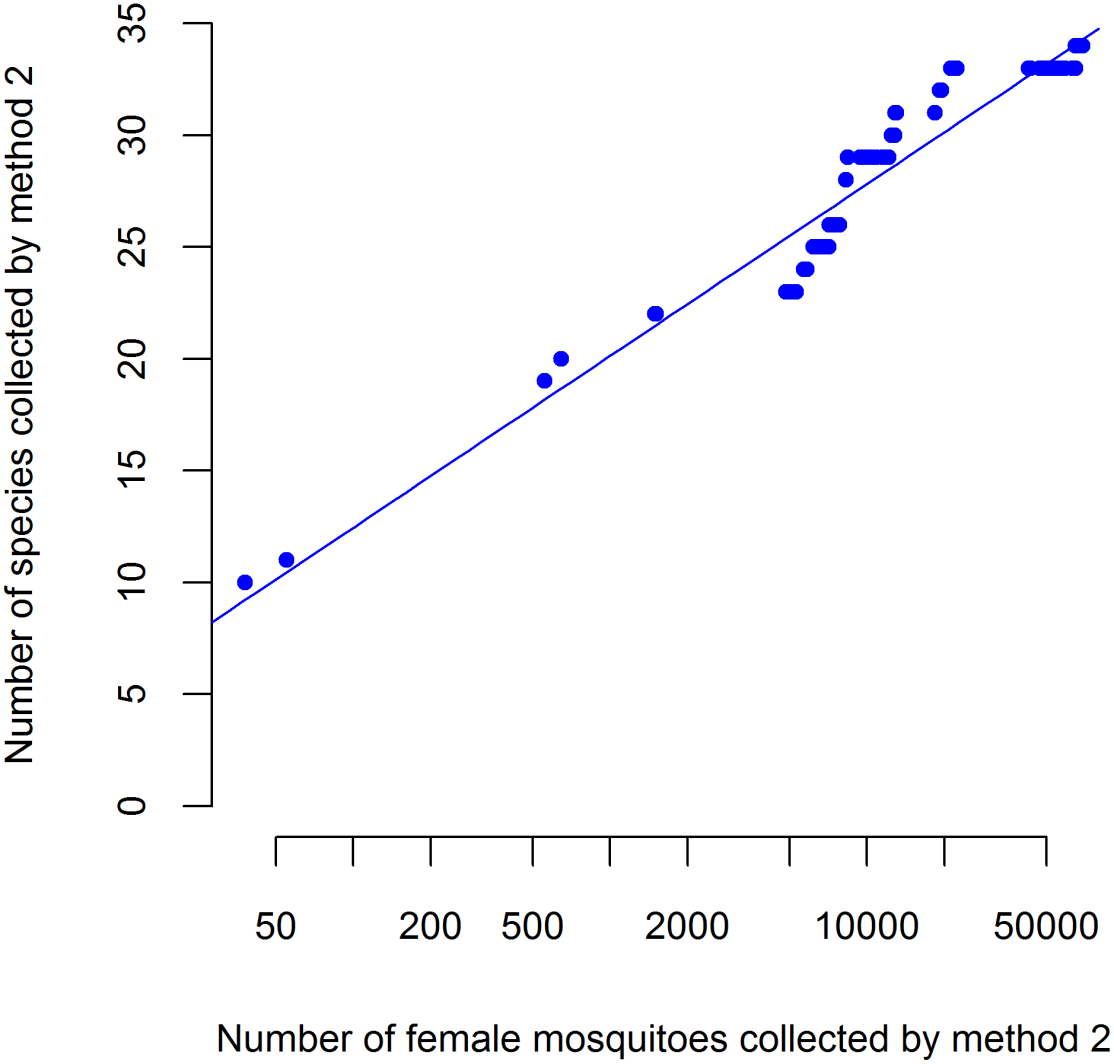
Number of species collected as a function of number of individual females collected by light traps near hosts (method 2). Estimated by linear regression y = -2.93 + log(x)*3.34, *R*^*2*^ = 0.92, *F* = 1373 and *p* < 0.001.

All 36 species identified during the study were observed in the mosquito collection using method 2, compared with 24 species using method 1, which yielded 575 female mosquitoes. Using results from linear regression (Fig 1), we estimate that 18 species would have been expected in a sample of 575 individual mosquitoes (the total captured by method 1) using method 2. To collect 24 species by method 2, at least 3500 mosquitoes would be required (Fig 1). Indeed, despite more mosquitoes being collected by method 2, Hill’s diversity numbers H_1_ and H_2_ were both higher for method 1 (Table 2), indicating a greater diversity of species was sampled by method 1 than by method 2. *Culex tritaeniorhynchus* was observed to be common by both methods. The collection from method 1 contained five other common species- *Cx*. *pseudovishnui*, *Cx*. *gelidus*, *Ar*. *subalbatus*, *Cx*. *vishnui* and *Ar*. *kesseli* (Table 1). Three other common species were observed in collections from method 2- *An*. *peditaeniatus*, *Cx*. *gelidus*, and *Cx*. *pseudovishnui*, with the rest of the observed species each constituting less than 5% of the total sample.

**Table 2:** Hill’s diversity numbers by sampling method used.

Sampling method	H_0_	H_1_	H_2_
Method 1 (outdoor resting box)	17	8.6	6.1
Method 1 (outdoor BPD hop cage)	22	9.4	6.1
Method 2 (light traps near hosts)	36	4.2	2.4

H_0_ is equivalent to species richness, H_1_ is the natural exponential of the Shannon-Weiner diversity index and H_2_ is the reciprocal of Simpson’s index [62,63].

With respect to the two resting collection methods, 153 mosquitoes were collected using resting boxes and 422 using the BPD hop cage. H_1_ and H_2_ were similar between these methods (Table 2). Species collected by hop cage but not resting box included *Ae*. *lineatopennis*, *An*. *barbirostris*, *An*. *nigerrimus*, *Ar*. *kuchingensis*, *Cq*. *crassipes*, *Cx*. *whitmorei*, *Ma*. *annunlifera*. Species collected by resting box but not hop cage included *An*. *vagus*, *Cx*. *infula* and *Ma*. *indiana*.

### Comparison of common species relative abundance between method 1 and method 2 at the village level

**Fig 2:**
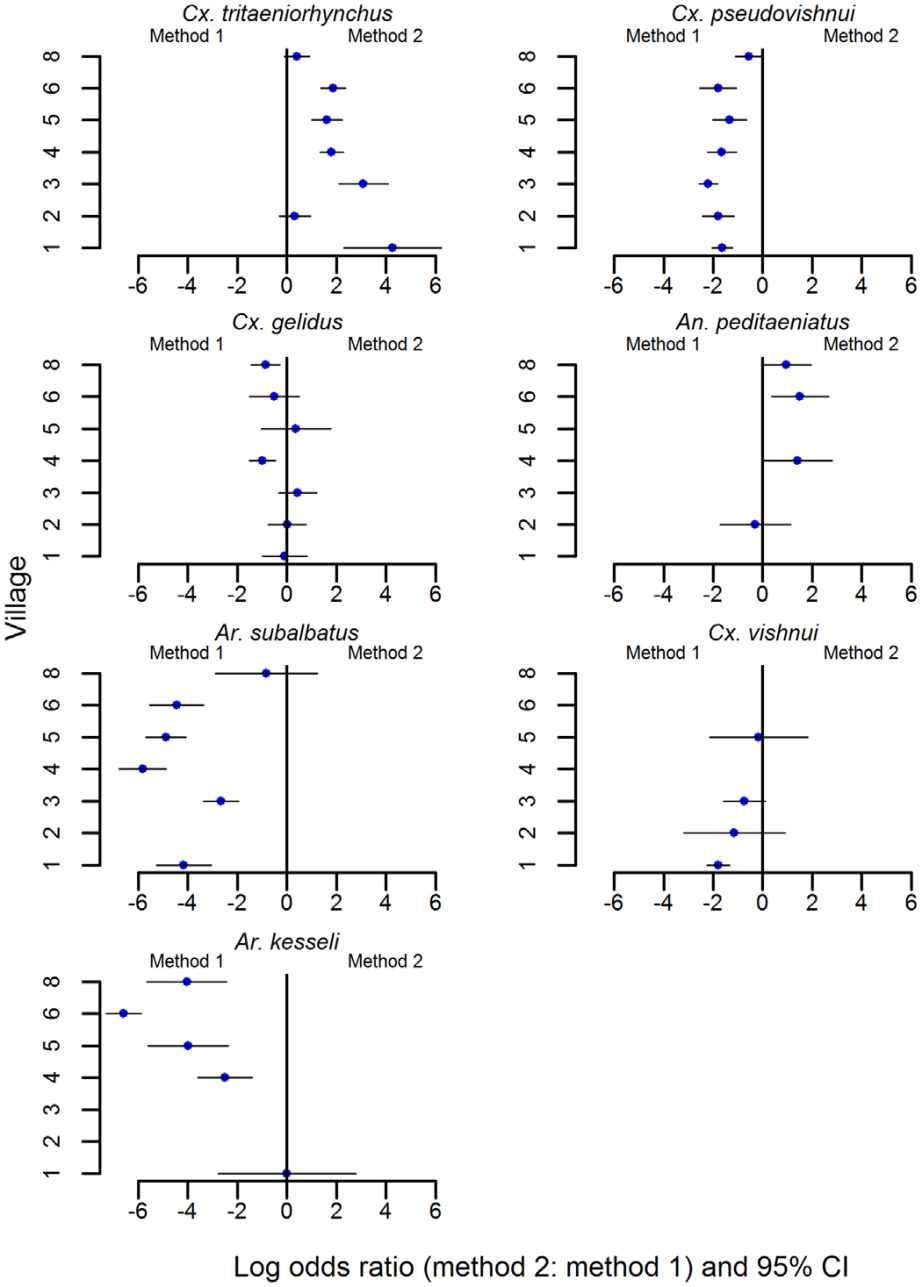
Comparison between sampling methods by village. Includes species constituting at least 5% of collections from method 1 (resting collection near oviposition sites) or method 2 (light trap collection near hosts) using log odds ratio tests. Confidence intervals that do not include zero indicate significant method bias. Absence of data for a species from a village indicates the species was not observed by one or both sampling methods.

In five of seven villages, *Cx*. *tritaeniorhynchus* was more likely to be selected from a collection using method 2 than from a collection using method 1 (Fig 2). There was no significant difference between sampling methods for this species in the 2^nd^ and 8^th^ villages visited. In the 8^th^ village there were no significant differences between sampling methods for *Cx*. *pseudovishnui*, *Ar*. *subalbatus* and *Ar*. *kesseli*. These three species were however more represented in the collection using method 1 than in the collection using method 2 for all other villages (Fig 2). There were no significant differences between sampling methods for *Cx*. *gelidus* and *Cx*. *vishnui* in the majority of villages (Fig 2).

### Effect of host community upon the relative abundance of common species in light traps

**Fig 3:**
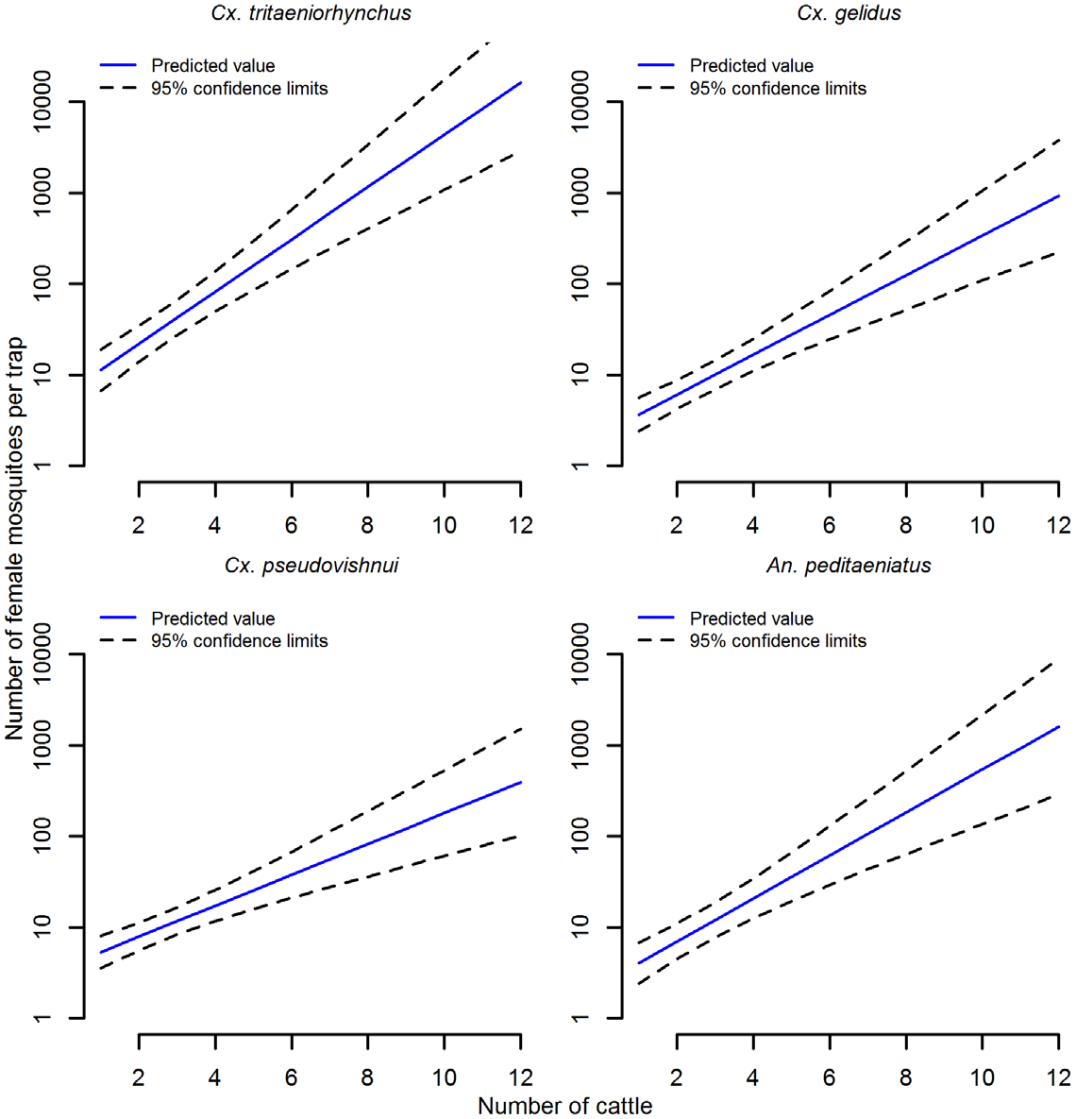
Prediction of the influence of cattle upon mosquito species relative abundance in light traps. Includes species constituting at least 5% of light trap collections. Associated linear regression results presented in S3 Table.

**Fig 4:**
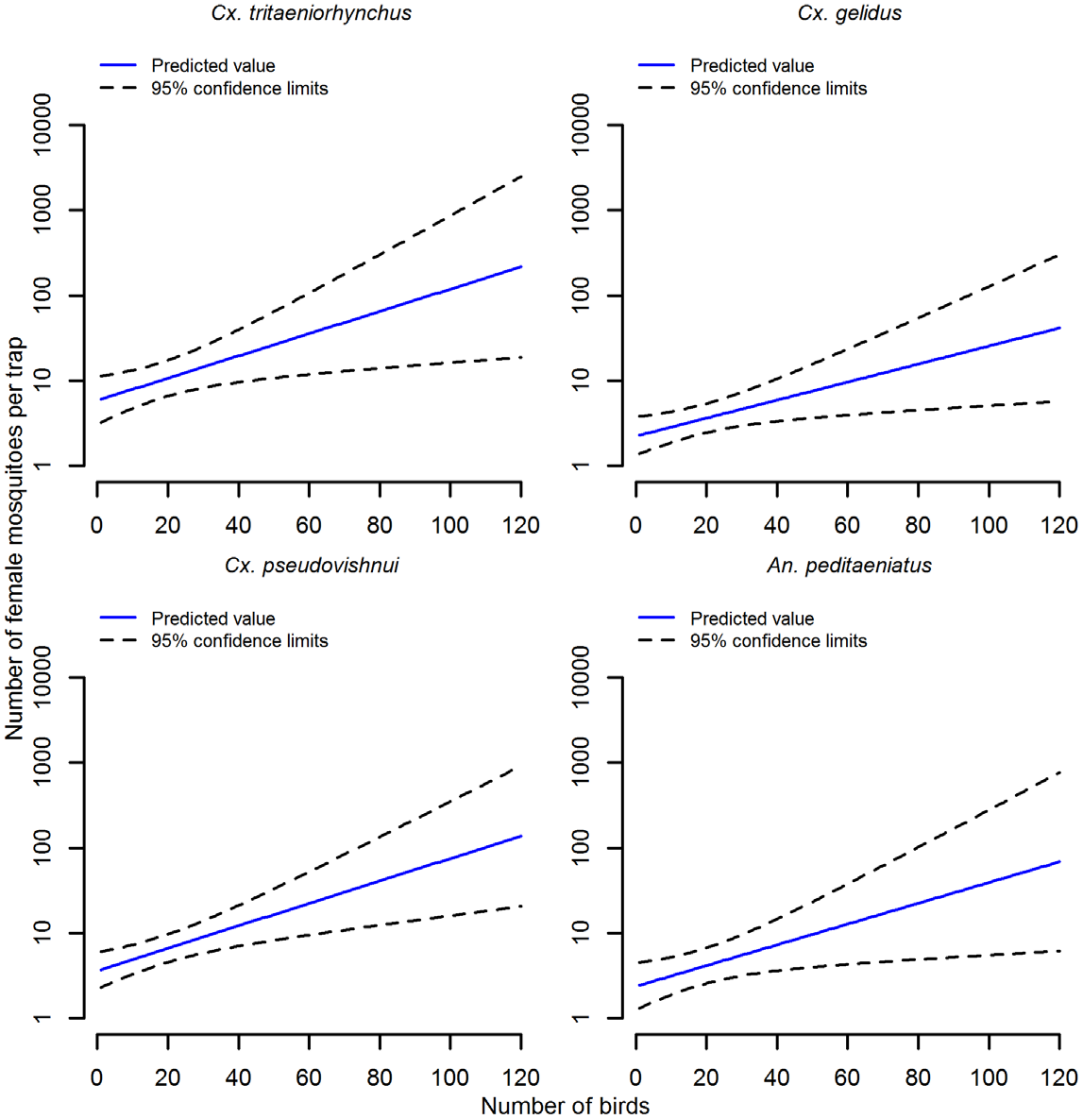
Prediction of the influence of birds upon mosquito species relative abundance in light traps. Includes species constituting at least 5% of light trap collections. Associated linear regression results presented in S3 Table.

**Fig 5:**
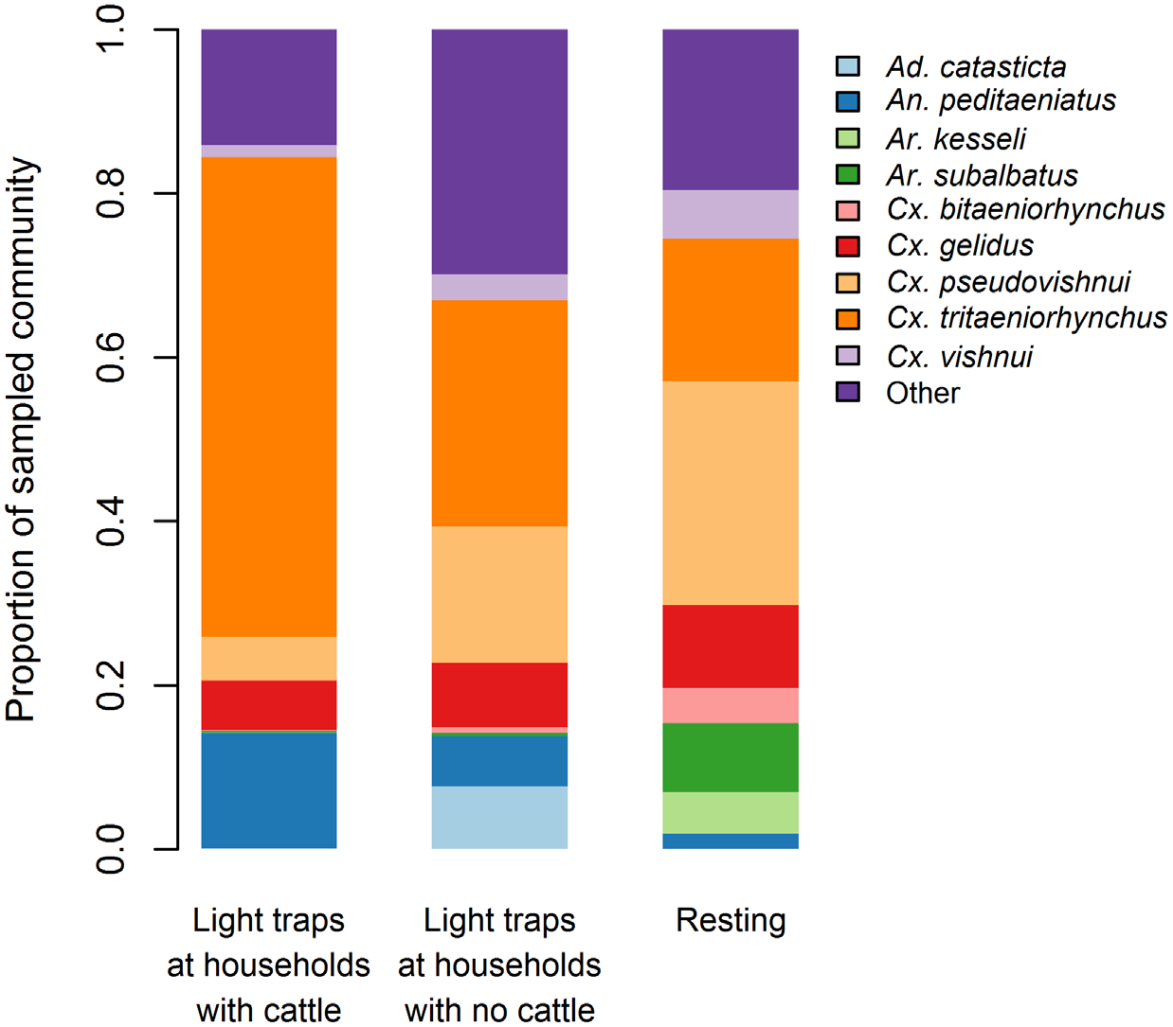
Species composition of samples from light traps in households with cattle, light traps in households with no cattle and resting collections, for villages where both light trap and resting collections were made.

Households had varying combinations of cattle, goats, ducks, chickens, pigeons and, rarely, geese or sheep. Pigs were only present in one of the eight villages. There was no association between the number of common mosquito species caught by light trap and the number of humans or goats in a household. Households with higher numbers of cattle on average had higher numbers of all four common species in light traps (Fig 3, S3 Table). The numbers of cattle in a household ranged from zero to 12. For each additional cow present in a household there was an approximate two-fold increase in the number of *Cx*. *tritaeniorhynchus* and approximately 1.5 to 1.7-fold increase of other common species (S3 Table). In households with no cattle, higher numbers of common mosquito species were more likely when there were higher numbers of birds in a household (Fig 4). The number of birds in a household ranged from zero to 120. In the absence of cattle, there was approximately a 1.3-fold increase in the numbers of each of the four common species for each additional 10 birds in a household (S3 Table). Though numbers of both birds and cattle influenced the relative abundance of all common mosquito species, the influence of cattle was higher for *Cx*. *tritaeniorhynchus* than the influence of birds relative to other mosquito species. The association between cattle numbers and abundance of individual mosquito species had implications for the mean relative abundance (Table 3) and species composition (Fig 5) by sampling method.

**Table 3:** Average light trap counts for common species (> 5% of the sample) according to presence or absence of cattle.

Species	Households with cattle			Households without cattle		
	Mean/ light trap	s.e.m.	Range	Mean/ light trap	s.e.m.	Range
*Culex tritaeniorhynchus*	415	139	0–12,866	24	11.6	0–230
*Anopheles peditaeniatus*	100	33	0–3183	5	2.1	0–28
*Culex gelidus*	43	12	0–1162	7	2.9	0–44
*Culex pseudovishnui*	38	8	0–722	15	5.9	0–103
*Culex vishnui*	10	4.6	0–405	3	2	0–41
*Armigeres subalbatus*	1	0.2	0–11	< 1	0.2	0–3
*Culex bitaeniorhynchus*	1	0.2	0–10	< 1	0.3	0–5
*Armigeres kesseli*	< 1	0.1	0–8	0	0	0
*Adeoymia catasticata*	< 1	0.3	0–28	7	6.7	0–140

s.e.m.- standard error of the mean

## Discussion

Entomological studies of potential JEV vectors in India have frequently collected mosquitoes from around cattle sheds at dusk and many report *Cx*. *tritaeniorhynchus* to constitute at least 50% of the sample [20–27,29–31,33,35,36,66]. This finding has been used as evidence supporting the theory that *Cx*. *tritaeniorhynchus* is the primary vector of JEV in this country [20–27,29–31,33,35–37,66].

In our study, while *Cx*. *tritaeniorhynchus* constituted the majority of the sample when using light traps in households with cattle, in households without cattle *Cx*. *tritaeniorhynchus* did not constitute the majority of the sample, nor from resting collections near oviposition sites. Our findings are consistent with a study from India where *Cx*. *tritaeniorhynchus* was two to forty times more abundant in cattle sheds at dusk than other species whereas during daytime resting collections other species, including *Cx*. *quinquefasciatus*, *Cx*. *pseudovishnui* and *Anopheles* spp, were more abundant [66]. Studies aiming to implicate vector species in transmission of JEV in areas where the transmission ecology differs substantially from that first described in Japan (accompanying article), should consider focusing catches around hosts able to transmit JEV instead of collections near dead-end hosts, such as cattle.

Other species, including *Cx*. *gelidus*, *Cx*. *quinquefasciatus*, *Ar*. *subalbatus* and anophelines have been observed to be infected with JEV in the wild [22,67,68]. Species in addition to *Cx*. *tritaeniorhynchus*, including *Ar*. *subalbatus*, *Cx*. *quinquefasciatus* and *Cx*. *pseudovishnui*, are capable of transmitting JEV under experimental conditions [67,69]. The relative contributions of other competent mosquito species to JEV transmission should be further investigated.

Differences in the relative abundance of competent species in collections from cattle using aspirators or light traps at dusk may be a result of alternative host-seeking strategies between species rather than actual differences in population density. Light traps in particular are biased because they capture phototactic species active after dark. *Culex pseudovishnui*, *Ar*. *subalbatus* and *Ar*. *kesseli* were significantly better represented in daytime resting collections near oviposition sites than light trap collections near hosts. During our surveys in Bangladesh, *Ar*. *subalbatus* was frequently observed during daylight hours. Similarly, Das *et al*. [70], which was one of few studies in India to undertake human-bait collections during daylight hours, found *Ar*. *subalbatus* to constitute 76% of the sample whilst the *Cx*. *vishnui* subgroup including *Cx*. *tritaeniorhynchus* constituted less than 5%. It is likely that light traps are not effective for catching this species because host-seeking females are most active before light traps become effective. This may also be the case for *Cx*. *pseudovishnui*, as Bhattacharyya *et al*.[71] reported the initial peak in biting for this species to occur at 19.00 compared with 21.00 for *Cx*. *tritaeniorhynchus*. The study was undertaken between May and June, when sunset times ranged from 17.45 to 18.10. The initial peak biting time for *Cx*. *pseudovishnui* was approximately one hour after sunset and for *Cx*. *tritaeniorhynchus* three hours after sunset. It is therefore possible that it may not be dark enough one hour after sunset for light traps to be fully effective for attracting *Cx*. *pseudovishnui*.

Indoor resting collections were not conducted during the current study and we may therefore have under-sampled species which tend to rest indoors including *Cx*. *quinquefasciatus*, *An*. *vagus* and *An*. *subpictus* [32]. Thus, these species may constitute a greater proportion of the true mosquito community in the study area than our methods account for. We also acknowledge that the current study represents a snapshot of mosquito relative abundance, with fieldwork being conducted for only three months of the year (Sep—Dec) and in a relatively small number of villages. Due to the political situation during the survey period we were unable to spend an equal amount of time in each village, resulting in variation in the amount of time for resting collections and number of light traps set in each village. While this sample is sufficient to demonstrate that there are important differences between sampling methods we cannot therefore account for potential variation in mosquito community composition in space and time.

Studies aiming to implicate vector species in JEV transmission should carefully consider potential method biases when estimating relative abundance. Initial studies in Japan [72] used traps baited with host species known to be able to transmit JEV, and those that were present in high density, to sample the mosquito community feeding on hosts of relevance to transmission rather than dead-end hosts such as cattle. Because cattle do not produce viremia sufficient to infect mosquitoes, they have the potential to dilute transmission, especially when they are present in high density relative to other hosts, as in regions of India and Bangladesh. This may in turn reduce the role of *Cx*. *tritaeniorhynchus* as a vector species given its preference for cattle [50]. Given that *Cx*. *tritaeniorhynchus* is not as abundant in samples using method 1, is an indicator that other mosquito species may be just as, or more abundant and should not be discounted as potential vectors. Where possible, supplementing resting collections with traps baited with hosts able to transmit JEV would assist in implicating mosquito species in transmission.

During our study, substantially more mosquitoes were required to be collected by light trap near hosts than by resting collection near oviposition sites in order to observe an equivalent number of species. As the most time consuming aspect of JEV- associated entomological fieldwork is often the identification of mosquitoes to species level, we suggest resting collection near oviposition sites to be more efficient for initially establishing the mosquito species present in an area because fewer individual mosquitoes would need to be identified in order to obtain a sample of the species present. This approach also requires relatively little equipment and does not require use of animal baits or electricity. Furthermore, because resting mosquitoes are often blood-fed, the same samples could be used for estimating the proportion of bloodmeals taken from different host species.

The total number of JEV isolates from *Cx*. *tritaeniorhynchus*, often obtained from sampling near cattle at dusk, has additionally been used as evidence that *Cx*. *tritaeniorhynchus* is the primary vector [37]; however, the number of JEV isolates from any mosquito species is a product of both the abundance of infected mosquitoes of that species and the probability that an individual of that species will be caught by the particular trapping method used. As *Cx*. *tritaeniorhynchus* is collected in large numbers compared with other species near cattle at dusk with light traps or aspirators, it may be expected that the highest number of viral isolates would also be obtained from this species using this method, regardless of their actual importance in transmitting JEV to humans. In this study, *Cx*. *tritaeniorhynchus* was approximately 11 times more abundant in light traps at households with cattle than *Cx*. *pseudovishnui*. This apparent difference in abundance may result from differences in the true abundance of the species, differences in trap efficiency, or both. Given the difference in the number of individuals caught, however, the prevalence of JEV in *Cx*. *pseudovishnui* would have to be at least 11 times greater than that for *Cx*. *tritaeniorhynchus* in order to be likely to yield a higher number of viral isolates from this species using collections near cattle. We advise that maximum likelihood estimation of virus prevalence in mosquito populations be considered alongside total number of viral isolates, to more accurately characterize the potential role of mosquito species.

Maximum likelihood estimates of prevalence would be more useful than total numbers, for implicating species important in maintaining JEV transmission, but additional studies are also required to identify species which present the highest risk of transmission to humans. The numbers of infectious bites on humans is related not only to the prevalence of virus in mosquitoes and the number of mosquitoes relative to hosts, but also mosquito host feeding patterns. Even if *Cx*. *tritaeniorhynchus* is the most important vector for maintaining JEV transmission, bloodmeal studies have shown *Cx*. *tritaeniorhynchus* to feed between 2 and 4% on humans [48,73]. In addition, human landing catches in India resulted in a sample of 2836 *Ar*. *subalbatus*, 579 *Cx*. *quinquefasciatus*, 41 *Cx*. *vishnui*, 29 *Cx*. *pseudovishnui* and no *Cx*. *tritaeniorhynchus*, in addition to species of *Anopheles* and *Mansonia* [70]. The species most important for human transmission in South Asia remain to be identified.

In addition to relative abundance, greater focus should be placed upon mosquito species feeding patterns, fine-scale variation in host and mosquito species community composition, and competence of hosts and vectors for viral replication in regions experiencing JE outbreaks where the transmission context is different from that first described in Japan (accompanying article).

## Supporting Information

S1 Fig Locations of villages in Bangladesh subject to entomological survey. (TIFF)

S1 Table Details of mosquito collections by village. (DOCX)

S2 Table Mosquito species comprising less than 5% of resting collection near oviposition sites (method 1) or light trap near hosts (method 2). (DOCX)

S3 Table Multiple linear regression analyzing the number of hosts in a household (n = 123) as explanatory variables for the log (x + 1) number of mosquitoes caught in light traps, for the four most common species by light trap. (DOCX)
