# Supplementary figures and images for: Sampling Design Influences the Observed Dominance of Culex tritaeniorhynchus: Considerations for Future Studies of Japanese Encephalitis Virus Transmission
Source: PLoS Negl Trop Dis. 2016 Jan 4;10(1):e0004249. doi: 10.1371/journal.pntd.0004249 (PMC4699645; doi:10.1371/journal.pntd.0004249)

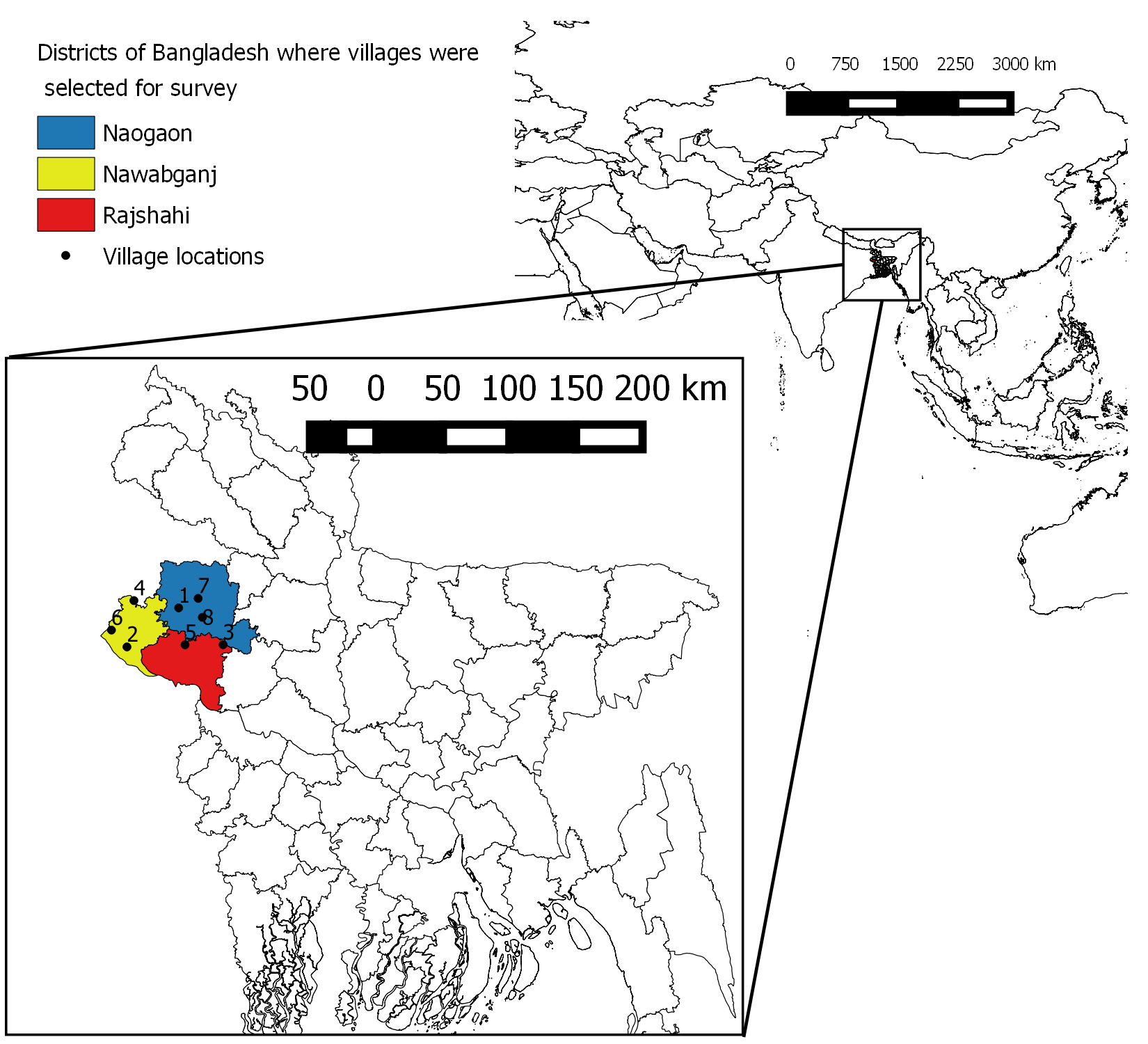

Supplement: S1 Fig — (TIFF) [file pntd.0004249.s001.tiff]
