# Supplementary material for: Sampling Design Influences the Observed Dominance of Culex tritaeniorhynchus: Considerations for Future Studies of Japanese Encephalitis Virus Transmission
Source: PLoS Negl Trop Dis. 2016 Jan 4;10(1):e0004249. doi: 10.1371/journal.pntd.0004249 (PMC4699645; doi:10.1371/journal.pntd.0004249)
Supplement: S1 Table — (DOCX) [file pntd.0004249.s002.docx]

**Table S1 Details of mosquito collections by village**

| **Village*** | **Number of resting collection days** | **Duration of time spent undertaking resting collections (hrs)** | **Number of light trap nights** | **Total number of light traps set/ village** |
| --- | --- | --- | --- | --- |
| 1 | 4 | 6.2 | 3 | 18 |
| 2 | 1 | 2.9 | 2 | 16 |
| 3 | 3 | 9.1 | 2 | 15 |
| 4 | 2 | 4.1 | 2 | 16 |
| 5 | 2 | 2.8 | 2 | 14 |
| 6 | 2 | 3.7 | 2 | 16 |
| 7 | 0 | 0 | 1 | 12 |
| 8 | 2 | 3.4 | 2 | 16 |

* Each village was surveyed only once for the number of nights given in the table
