# Supplementary material for: Sampling Design Influences the Observed Dominance of Culex tritaeniorhynchus: Considerations for Future Studies of Japanese Encephalitis Virus Transmission
Source: PLoS Negl Trop Dis. 2016 Jan 4;10(1):e0004249. doi: 10.1371/journal.pntd.0004249 (PMC4699645; doi:10.1371/journal.pntd.0004249)
Supplement: S2 Table — (DOCX) [file pntd.0004249.s003.docx]

**Table S2 Mosquito species comprising less than 5% of resting collection near oviposition sites (method 1) or light trap near hosts (method 2)**

| **Species** | **Total number caught using method 1** | **% of sample from method 1** | **95 % confidence interval** | | **Total number caught using method 2** | **% of sample from method 2** | **95 % confidence interval** | |
| --- | --- | --- | --- | --- | --- | --- | --- | --- |
| *Culex* sp. | 11 | 1.9 | 0.8 | 3.0 | 2006 | 2.7 | 2.6 | 2.8 |
| *Culex Culex vishui* group | 13 | 2.3 | 1.1 | 3.5 | 1913 | 2.6 | 2.5 | 2.7 |
| *Anopheles Anopheles nigerrimus* | 3 | 0.5 | 0.0 | 1.1 | 1661 | 2.2 | 2.1 | 2.3 |
| *Aedes Neomelaniconion lineatopennis* | 17 | 3.0 | 1.6 | 4.4 | 931 | 1.3 | 1.2 | 1.3 |
| *Anopheles* sp. | 1 | 0.2 | 0.0 | 0.5 | 877 | 1.2 | 1.1 | 1.3 |
| *Mansonia Mansonioides uniformis* | 10 | 1.7 | 0.7 | 2.8 | 607 | 0.8 | 0.8 | 0.9 |
| *Anopheles Cellia vagus* | 2 | 0.4 | 0.0 | 0.8 | 444 | 0.6 | 0.5 | 0.7 |
| *Culex Culex infula* | 1 | 0.2 | 0.0 | 0.5 | 399 | 0.5 | 0.5 | 0.6 |
| *Anopheles Anopheles barbirostris* | 1 | 0.2 | 0.0 | 0.5 | 347 | 0.5 | 0.4 | 0.5 |
| *Culex Culex sinensis* | 6 | 1.0 | 0.2 | 1.9 | 229 | 0.3 | 0.3 | 0.4 |
| Unidentifiable | 0 | 0.0 | 0.0 | 0.0 | 212 | 0.3 | 0.3 | 0.3 |
| *Mansonia Mansonioides indiana* | 1 | 0.2 | 0.0 | 0.5 | 202 | 0.3 | 0.2 | 0.3 |
| *Aedeomyia Aedeomyia catasticta* | 0 | 0.0 | 0.0 | 0.0 | 199 | 0.3 | 0.2 | 0.3 |
| *Anopheles Cellia annularis* | 0 | 0.0 | 0.0 | 0.0 | 195 | 0.3 | 0.2 | 0.3 |
| *Mansonia Mansonioides annulifera* | 1 | 0.2 | 0.0 | 0.5 | 181 | 0.2 | 0.2 | 0.3 |
| *Anopheles Cellia varuna* | 0 | 0.0 | 0.0 | 0.0 | 168 | 0.2 | 0.2 | 0.3 |
| *Culex Culex bitaeniorhynchus* | 25 | 4.4 | 2.7 | 6.0 | 113 | 0.2 | 0.1 | 0.2 |
| *Culex Culex epidesmus* | 1 | 0.2 | 0.0 | 0.5 | 104 | 0.1 | 0.1 | 0.2 |
| *Culex Culex fuscocephala* | 11 | 1.9 | 0.8 | 3.0 | 94 | 0.1 | 0.1 | 0.2 |
| *Culex Culex hutchinsoni* | 10 | 1.7 | 0.7 | 2.8 | 39 | 0.1 | 0.0 | 0.1 |
| *Anopheles Cellia subpictus* | 0 | 0.0 | 0.0 | 0.0 | 36 | 0.0 | 0.0 | 0.1 |
| *Armigeres* sp. | 1 | 0.2 | 0.0 | 0.5 | 33 | 0.0 | 0.0 | 0.1 |
| *Culex Culex quinquefasciatus* | 0 | 0.0 | 0.0 | 0.0 | 31 | 0.0 | 0.0 | 0.1 |
| *Coquillettidia Coquillettidia crassipes* | 1 | 0.2 | 0.0 | 0.5 | 21 | 0.0 | 0.0 | 0.0 |
| *Anopheles Cellia tessellatus* | 0 | 0.0 | 0.0 | 0.0 | 15 | 0.0 | 0.0 | 0.0 |
| *Aedes* sp. | 0 | 0.0 | 0.0 | 0.0 | 14 | 0.0 | 0.0 | 0.0 |
| *Mansonia* sp. Unidentified | 0 | 0.0 | 0.0 | 0.0 | 7 | 0.0 | 0.0 | 0.0 |
| *Mansonia* sp. | 0 | 0.0 | 0.0 | 0.0 | 5 | 0.0 | 0.0 | 0.0 |
| *Mimomyia* sp. | 5 | 0.9 | 0.1 | 1.6 | 5 | 0.0 | 0.0 | 0.0 |
| *Armigeres Armigeres kuchingensis* | 1 | 0.2 | 0.0 | 0.5 | 4 | 0.0 | 0.0 | 0.0 |
| *Aedes Stegomyia albopictus* | 15 | 2.6 | 1.3 | 3.9 | 3 | 0.0 | 0.0 | 0.0 |
| *Uranotaenia Uranotaenia rampae* | 0 | 0.0 | 0.0 | 0.0 | 3 | 0.0 | 0.0 | 0.0 |
| *Culex Lutzia fuscanus* | 0 | 0.0 | 0.0 | 0.0 | 2 | 0.0 | 0.0 | 0.0 |
| *Mansonia annulata* | 0 | 0.0 | 0.0 | 0.0 | 2 | 0.0 | 0.0 | 0.0 |
| *Aedes Aedimorphus vexans* | 0 | 0.0 | 0.0 | 0.0 | 1 | 0.0 | 0.0 | 0.0 |
| *Culex Culex whitmorei* | 1 | 0.2 | 0.0 | 0.5 | 1 | 0.0 | 0.0 | 0.0 |

Unconfirmed species were specimens of the same morphological type but that were not described in any of the taxonomic keys.
