# Supplementary material for: Sampling Design Influences the Observed Dominance of Culex tritaeniorhynchus: Considerations for Future Studies of Japanese Encephalitis Virus Transmission
Source: PLoS Negl Trop Dis. 2016 Jan 4;10(1):e0004249. doi: 10.1371/journal.pntd.0004249 (PMC4699645; doi:10.1371/journal.pntd.0004249)
Supplement: S3 Table — (DOCX) [file pntd.0004249.s004.docx]

**S3 Table**

**Multiple linear regression analyzing the number of hosts in a household (n=123) as explanatory variables for the log (x + 1) number of mosquitoes caught in light traps, for the four most common species by light trap.**

|  |  |  |  | **Cattle:bird** | | **Cattle** | | **Bird** | |
| --- | --- | --- | --- | --- | --- | --- | --- | --- | --- |
| **Species** | ***R^2^*** | ***F*** | ***p*** | ***p*** | **Coef.** | ***p*** | **Coef.** | ***p*** | **Coef.** |
| *Culex tritaeniorhynchus* | 0.35 | 22.98 | <0.001 | 0.0015 | -0.008 | <0.001 | 0.66 | 0.012 | 0.03 |
| *Culex gelidus* | 0.29 | 17.51 | <0.001 | <0.001 | -0.007 | <0.001 | 0.5 | 0.013 | 0.02 |
| *Culex pseudovishnui* | 0.23 | 11.54 | <0.001 | <0.001 | -0.007 | <0.001 | 0.39 | 0.001 | 0.03 |
| *Anopheles peditaeniatus* | 0.28 | 16.66 | <0.001 | <0.001 | -0.006 | <0.001 | 0.54 | 0.018 | 0.02 |

Numbers of cattle, birds, humans and goats including interaction terms, were initially included in the model, and then a step-wise elimination of non-significant terms according to the F-test was undertaken to achieve the minimal adequate model. The minimal adequate model for all species included the number of cattle, number of birds and an interaction between the two.
